# Prognostic Role of MicroRNAs in Human Non-Small-Cell Lung Cancer: A Systematic Review and Meta-Analysis

**DOI:** 10.1155/2018/8309015

**Published:** 2018-10-21

**Authors:** Shree Ram Lamichhane, Thanuja Thachil, Paolo De Ieso, Harriet Gee, Simon Andrew Moss, Natalie Milic

**Affiliations:** ^1^College of Health and Human Sciences, Charles Darwin University, Alan Walker Cancer Care Centre, Darwin, NT, Australia; ^2^Department of Radiation Oncology, Crown Princess Mary Cancer Centre, Westmead Hospital and School of Medicine, University of Sydney, Australia

## Abstract

**Background:**

MicroRNAs (miRNAs) have been found to play an important role in the development and outcomes for multiple human cancers. Their role as a prognostic biomarker in non-small-cell lung cancer (NSCLC) remains unclear. This meta-analysis aims to clarify the role of various miRNAs in the survival of NSCLC patients.

**Materials and Methods:**

All studies were identified through medical database search engines. A meta-analysis was conducted to assess the correlation between miRNAs expressions and overall survival among those NSCLC studies. Relevant data were extracted from each eligible study regarding baseline characteristics and key statistics such as hazard ratio (HR), 95% confidence interval (CI), and *P* value, which were utilized to calculate a pooled effect size.

**Result:**

Thirty-two studies were included in the meta-analysis. Using a random effect model, the combined HR and 95% CI for overall survival (OS) was calculated as 1.59 (1.39–1.82), predicting a poor overall survival. Five miRNAs (miR-21, miR-155, miR-*let*-7, miR-148a, and miR-148b) were found to be of significance for predicting OS in at least two studies, hence, selected for subgroup analysis. Subgroup analysis disclosed that elevated levels of miR-21 and miR-155 in both cancer tissue and blood samples were associated with worse OS. Compared to American studies (I-squared: <0.001% and *P* value: 0.94), Asian and European studies exhibited greater heterogeneity in miRNA expression and relationship to OS (I-squared, *P* values were approximately 78.85%, <0.001 and 61.28%, 0.006, respectively). These subgroup analyses also highlighted that elevated expression of miR-21 and miR-155 and low levels of expression of miR-148a, miR-148b, and miR-*let*-7 were associated with poor prognosis in NSCLC.

**Conclusion:**

miR-21, miR-155, miR-148a, miR-148b, and miR-*let*-7 are consistently up- or downregulated in NSCLC and are associated with poor OS. These miRNAs show potential as useful prognostic biomarkers in the diagnosis, treatment, and follow-up of NSCLC.

## 1. Introduction

Lung cancer is the most common cause of death from cancer worldwide with a bleak overall 5-year survival rate of less than 15% for all-comers [1]. For treatment purposes, lung cancer is divided into two major subgroups, small cell (SCLC) and non-small-cell lung cancer (NSCLC). NSCLC accounts for approximately 80% of all lung cancers and is further divided into three major histological subtypes, which are adenocarcinoma, squamous cell carcinoma, and large-cell carcinoma [1, 2]. NSCLC is often diagnosed at an advanced stage when the prognosis is poor, resulting in low survival rates despite recent improvements in treatments [3]. Detection of accurate biomarkers can prognosticate cancer correctly with an aim to improve overall survival (OS). There is increasing evidence to suggest that micro-ribonucleic acids (miRNAs) play a critical role in the development of NSCLC and have been proposed as potential biomarkers for NSCLC prognosis and response to therapy [3, 4].

miRNAs are small (~22 nucleotides) noncoding RNAs that regulate more than half of the genes in human cells and are associated with various biological activities including cell proliferation, cell differentiation, cell migration, disease initiation, disease progression, and apoptosis [5]. miRNAs modulate gene activity at the posttranscriptional level by degrading or inhibiting the translation of their messenger RNA (mRNA) targets. It has been observed that miRNAs expression is frequently upregulated for oncogenic miRNA and downregulated for tumor suppressor miRNA [6].

Many investigators have carried out miRNA profiling studies in NSCLC with cell lines, paired tissue samples, and blood samples. Microarray-based miRNA profiling assays are robust methods of screening hundreds of miRNAs. Given a large number of potential candidate miRNAs, well-established miRNA signatures documented in the literature have been identified [4, 7–11]. In collating the results and verifying miRNA profile platforms, a major challenge is normalization and determining significant thresholds.

A pooled analysis of multiple miRNA expression profile studies for NSCLC patients was performed to explore the association between miRNA expressions and OS. The starting point of this meta-analysis was to collect various published miRNA profiling studies comparing microRNA expressions in NSCLC patients and correlating high expression levels compared to low expression profiles against OS. By extracting summary statistics from these studies for survival endpoints, we analysed the predictive value of miRNA-148a (miR-148a), miRNA-148b (miR-148b), miRNA-let-7 (*Let*-7), miRNA-21 (miR-21), and miRNA-155 (miR-155) for NSCLC prognosis.

## 2. Materials and Methods

This meta-analysis was performed following the guidelines of the preferred reporting items for systematic reviews and meta-analysis (PRISMA) statement [12].

### 2.1. Search Strategy

The scientific literature published from January 2004 to March 2017 was interrogated using 5 different search engines: Scopus, PubMed, Science Direct, Web of Science, and Medline using key search words, including “microRNA expression or miRNA expression,” “lung cancer or NSCLC,” “prognosis,” “radiotherapy,” “radioresistance,” “radiosensitivity,” and “Human” (Supplementary Table 1). A manual review of references from published articles was also performed to select some additional studies.

### 2.2. Study Selection

PubMed search engine was selected as it provided the most relevant literature search for this topic. We reviewed all titles, including abstracts and full texts and ensured adherence to the inclusion and exclusion criteria for the meta-analysis. The primary literature that contained expression profiles of various miRNAs with multivariate analysis (high vs. low) and information including OS, HRs, 95% CIs, and *P* values were included in this meta-analysis. Moreover, included studies were also required to report on clinicopathological and demographic data associated with the patient's samples. Studies were excluded if they did not describe the association between miRNA expression and OS. Articles without full text or written in a language other than English were also excluded. Any doubt or uncertainty regarding the quality and inclusion of a piece of research work was resolved with the consensus of two clinical oncologists.

### 2.3. Quality Assessment

Two reviewers critically assessed the quality of all the studies included in this meta-analysis. All the studies were categorised into three groups: “unsatisfactory,” “satisfactory,” and “good” quality. The cut-off score was designed so that each study needed to be above “satisfactory” as described in Table 1.

### 2.4. Data Extraction

We independently extracted the required information from all eligible studies for meta-analysis. Prespecified data parameters included:

1. Demographic data regarding population, ethnicity, and survival rates during follow-up
2. Tumor data (histology, stage, primary lesions, and lymphoid node invasion)
3. Experimental data involving study design, materials, assays, and dysregulation of miRNAs expression
4. Statistical data including HRs for OS, 95% CI, and *P* value
5. Publication data (author's name, publication year, and journal title)

### 2.5. Statistical Analysis

HRs and 95% CIs extracted from the graphical survival plots from eligible articles were combined for the OS results. Forest plots were used to illustrate the association of miRNAs expression and OS. Heterogeneity was assessed using the Cochran Q test and Higgins I-squared statistic. *P* value less than 0.05 (*P* < 0.05) and I-squared value greater than 50% (I-square > 50%) indicated the presence of significant heterogeneity across studies. The random effect model was applied in the presence of heterogeneity between studies. An observed HR > 1 indicated a worse OS and poor prognosis in the group with elevated or reduced miRNA expressions. Publication bias was evaluated with the inverted funnel plot and the Egger's and Begg's bias indicator test. All the *P* values were two-sided, with *P* value less than 0.05 (*P* < 0.05) considered statistically significant. All calculations were performed using Comprehensive Meta-Analysis Version 3.0 software (Biostat, USA).

## 3. Results

### 3.1. Literature Search and Study Characteristics

**Figure 1:**
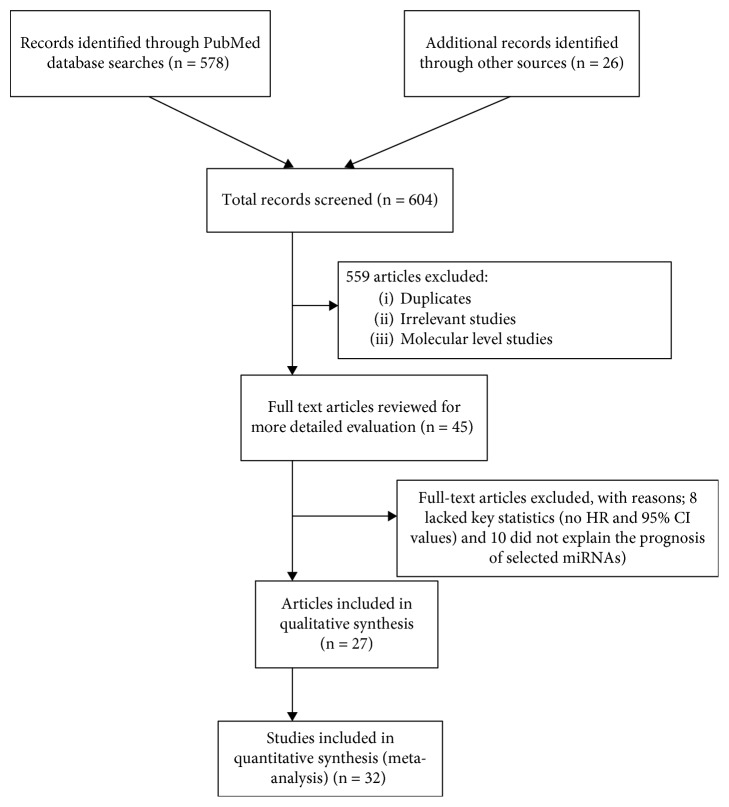
Flow chart of literature review and study selection process.

A preliminary online PubMed search highlighted a total of 578 studies concerning miRNA expression and lung cancer prognosis. An additional 26 studies were included from references and citations within the primary highlighted articles. Total 559 records were excluded as they represented irrelevant studies to the current analysis, review articles, letters, and *in vitro/in vivo* studies. Based on the readings of the article titles and abstracts and according to the inclusion and exclusion criteria, 45 articles were selected for more detailed evaluation. An additional 18 articles were excluded as they lacked key statistics in which eight articles did not mention HR or 95% CI values, whereas ten articles did not report on OS of the selected miRNAs. Finally, a total of 27 articles consisting of 32 independent studies were included in the meta-analysis. A flow chart of the study selection process is shown in Figure 1. Three articles [13–15] included two independent cohorts each, whereas one article [16] included three independent cohorts.

The data from the 32 studies included in the meta-analysis is summarized in Table 2. There was a total of 5553 samples from all of the studies, which was sorted according to country: United States (1439), Europe (662), and Asia (3452). Most Asian patient samples were from China (81.55%). Twenty-eight retrospective studies included tissue samples while four prospective studies used liquid biopsy samples [13, 17, 18]. miRNA expression levels were predominantly studied in paired tumor tissues, i.e., cancer tissue and adjacent benign tissue (20 out of 28 studies) as eight tissue sample studies made no mention if patient samples were paired tissue samples or single tumor biopsies. Among all 28 tissue sample studies, 12 studies included all tumor subtypes, whereas eight studies included only adenocarcinoma, four studies included only squamous cell carcinoma, and four studies [13, 19–21] did not differentiate the tumor subtype.

All 32 studies reported the prognostic value of 21 different miRNAs explaining OS. The upregulated and downregulated miRNAs reported in all studies are listed in Table 3. Twenty-eight studies reported on tumors at various stages of clinical presentation, whereas this information was absent in seven studies [13–15, 20, 21]. *In situ* hybridization (ISH) was utilized in four studies [15, 22, 23], while miRNA array and quantitative real-time PCR (qRT-PCR) remained the predominant techniques for miRNA detection in the remaining studies.

### 3.2. Study Outcomes

**Figure 2:**
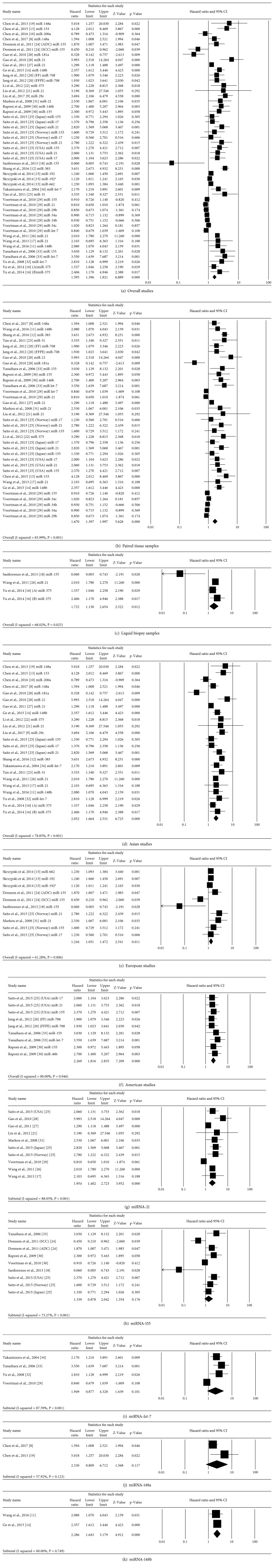
Forest plots of the studies that evaluated the hazard ratios of high vs. low miRNA expression. (a) Forest plot of the relationship between various miRNA expression and overall survival (OS) in NSCLC patients included in the meta-analysis. (b) Forest plot of the survival data reported in the studies with paired tissue samples (cancerous and adjacent noncancerous) as the source of miRNAs. (c) Forest plot of the survival data reported in the studies based on liquid biopsy samples as a source of miRNAs. (d) Forest plot of survival data from Asia. (e) Forest plot of survival data from Europe. (f) Forest plot of survival data from America. (g) Forest plot of the relationship between high miRNA-21 expression and overall survival in cancer patients with both random and fixed effects model. (h) Forest plot of the included studies that evaluated the hazard ratio of high miRNA-155 expression vs. low expression. (i) Forest plot of the relationship between lower miRNA-let-7 expression and OS in selected studies. (j) Forest plot of survival data for low miRNA-148a expression. (k) Forest plot of survival data for low miRNA-148b expression and OS in NSCLC studies.

All the included articles reported a close relationship between miRNAs and lung cancer prognosis. Among the 21 reported miRNAs, increased expression of miR-21, miR-155, miR-662, miR-708, miR-31, and miR-146b and decreased expression of miR-148a, miR-29c, miR-200a, miR-148b, miR-383, miR-153, miR-375, miR-155, miR-181a, and miR-*let*-7 were found associated with poor survival in NSCLC. Five miRNAs (miR-21, miR-155, miR-*let*-7, miR-148a, and miR-148b) were reported in at least two studies and provided all the key data to perform subgroup meta-analysis. The HRs and 95% CIs extracted from the studies were combined to interrogate the relationship between miRNA expression and lung cancer prognosis. The combined results revealed that both high and low expression levels of the listed miRNAs were associated with a poor prognosis in NSCLC, with a combined HR > 1.5. The combined HR (95% CI) for all 32 studies was calculated as 1.59 (1.39–1.82; *P* < 0.001), indicating a high level of heterogeneity (I-squared = 84.97%, *P* < 0.001). For all the data showing high heterogeneity, random effects model was applied (Figure 2).

### 3.3. Subgroup Analysis

Considering the heterogeneity among the studies, the effect of miRNA expression was further evaluated by subgroup analysis. The subgroups were classified according to the repeated miRNAs on the studies, which are the source of those miRNAs, miRNA assay methods, analysis type, and patient origin (Table 4). The association between miRNA expressions and OS outcome was statistically significant in most of the subgroups analysis including miRNA sources, sample origin, miRNA assay by qRT-PCR (HR = 1.05, 95% CI 0.58–1.87, *P* < 0.001), and univariate and multivariate analysis (HR = 2.29, 95% CI 1.02–5.12, *P* = 0.043 and HR = 1.54, 95% CI 1.35–1.76, *P* < 0.001, respectively) except ISH assay method (HR = 1.05, 95% CI 0.58–1.87, *P* = 0.870).

For all 20 studies using the paired tissue samples as patient's sample, the random effect model pooled HR for OS was calculated as 1.67 (95% CI: 1.39–1.99, *P* > 0.001), suggesting significant heterogeneity among the studies. Moreover, significant publication bias observed (Egger's test, *P* = 0.0001) among the included studies. High miRNA expression levels were observed in tumor tissues compared to normal adjacent tissue. The overexpression of miR-21 [16, 19, 20, 24–27], miR-155 [15, 16, 20, 28, 29], miR-662 [30], miR-708 [14], miR-31 [31], and miR-146b [28] predicted poor OS despite the cancer site. As eight studies did not specify if the patient's samples were taken as paired tissue sample or just single cancer tissue samples, these were excluded from paired tissue sample subgroup. Four studies targeted serum or plasma miRNAs, where both lower and higher expression levels of miRNAs were found associated with poor survival as well, with the combined HR 1.73 (95% CI: 1.13–2.65, *P* = 0.012). No apparent bias was observed (Egger's test, *P* = 0.151) among the studies.

Another subgroup analysis was performed for the location of sample collection to explore the cause of the heterogeneity between studies. The Asian and European subgroups exhibited greater heterogeneity (I-squared, *P* values approximately 78.85%, <0.001 and 61.28%, 0.006, respectively) compared to the American subgroup (I-squared: 0.00% and *P* value: 0.946). However, no significant publication bias was observed in the Asian and European studies (Egger's test, *P* = 0.125 and *P* = 0.397, respectively) compared to the American studies (Egger's test, *P* = 0.023). Overall, higher expression levels of miRNAs (miR-21, miR-155, miR-662, and miR-31) and lower expression levels of miRNAs (miR-148a, miR-29c, miR-200a, miR-148b, miR-181a, miR-153, miR-383, miR-375, and miR-*let*-7) reflected poorer prognosis. Particularly, the high expression level of miR-21, miR-155, miR-708, and miR-146b were correlated with poor overall survival in American studies.

Five miRNAs (miR-21, miR-155, miR-*let*-7, miR-148a, and miR-148b) were reported in at least two studies and were specifically analysed under subgroup analysis to evaluate the association between miRNA expression and overall survival in NSCLC.

#### 3.3.1. miR-21 Expression in NSCLC Prognosis

Eight articles (*n* = 2025) reported the effect of miRNA-21 on the prognosis of NSCLC patients (Figure 2(g)). Of these studies, seven provided overall survival data and one provided relapse-free survival data [16]. Evident heterogeneity was detected among all the studies (I-square = 88.05%, *P* < 0.001), suggesting the presence of other contributing factors. Overall, the random-effects model revealed that miRNA-21 expression was inversely associated with OS (HR: 1.95; 95% CI: 1.40–2.72; *P* < 0.001) in NSCLC patients. However, the asymmetry test (OS, Egger test, *P* = 0.227) indicated the existence of symmetry in the funnel plot which indicated no apparent bias in the studies included in the meta-analysis.

#### 3.3.2. miR-155 Expression in NSCLC Prognosis

Figure 2(h) displays the forest plot representation for the analysis of miRNA-155 and OS. Six articles (*n* = 2047) with nine individual cohort studies were subjected to the analysis. The random effect model was used to calculate the pooled effect size due to the presence of prominent heterogeneity among studies (I-square = 73.27%, *P* < 0.001). Elevated miRNA-155 expression was identified as a moderate predictor of poor OS regardless of the source of miRNAs. The pooled HR for OS was calculated as 1.33 (95% CI: 0.87–2.04, *P* = 0.176), suggesting no statistical significance. Even though significant interstudy heterogeneity was observed, the Egger test result indicated the presence of no significant publication bias (*P* = 0.242) among the studies.

#### 3.3.3. miR-*let*-7 Expression and NSCLC Prognosis

Four studies (*n* = 1239) evaluated the association between miR-*let*-7 expression in the tumor tissue samples and the prognosis of NSCLC patients, with two reporting OS [20, 21] and the other two with postoperative survivals [29, 32]. The adjusted HR was calculated as 1.94 (95% CI: 0.87–4.32) using random-effect model, suggesting prominent heterogeneity (I-square = 87.59%, *P* < 0.001). Moreover, significant publication bias observed among these selected studies (Egger's test, *P* = 0.010). The calculated high HR value concluded that downregulation of miR-*let*-7 in tumor tissue was associated with poor overall survival of the NSCLC patients among those studies.

#### 3.3.4. miRNA-148a and NSCLC Prognosis

Two studies (*n* = 377) suggested that low miRNA-148a expression levels were associated with poor survival in the patients with NSCLC [22, 33]. Evident interstudy heterogeneity was observed across these studies (I-square = 57.92%, *P* = 0.123). The pooled HR for OS was calculated as 2.33 (95% CI: 0.80–6.71, *P* = 0.117), suggesting a significant impact of miR-148a downregulation on the OS in NSCLC patients.

#### 3.3.5. miRNA-148b and NSCLC Prognosis

Two studies (*n* = 380) reported lower miR-148b expression as a predictor for poor OS in NSCLC patients using multivariate analysis [34, 35]. No significant heterogeneity was observed across these studies (OS, I-square = 0.00%, *P* = 0.749). Our analysis also revealed that the low miR-148b expression is associated with a significant poor prognosis. The pooled HR for OS was calculated as 2.28 (95% CI: 1.64–3.17, *P* < 0.001).

### 3.4. Publication Bias

**Figure 3:**
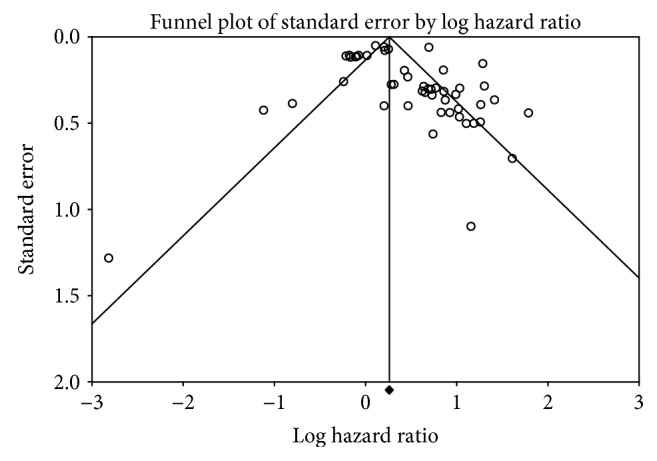
Funnel plots of studies included in the meta-analysis of NSCLC.

Finally, publication bias of the included studies was assessed by a Begg's funnel plot and Egger's test (Figure 3). The result of both Begg's test (*P* = 0.009) and Egger's test (*P* = 0.007) provided the statistical evidence of funnel plot asymmetry concluding apparent bias in the studies included in the meta-analysis.

### 3.5. Sensitivity Analysis

Sensitivity analysis was performed to evaluate whether the differences between studies induced instability in the meta-analysis or not. It was performed by sequential omission of individual studies using the fixed-effects model. No particular study was identified that influenced the overall results.

## 4. Discussion

We conducted a comprehensive systematic literature review to explore the utility of miRNA biomarkers that can be robustly evaluated in predicting prognosis in NSCLC patients. To our knowledge, this is the first extensive meta-analysis undertaken including the wider time frame (January 2004 to March 2017) and a wide range of miRNAs from both paired and liquid biopsy samples, and their subsequent ability to determine NSCLC prognosis. This meta-analysis pooled high-quality global studies concerning various miRNA expressions and cancer prognosis regarding OS.

Although miRNAs studied in the previous studies were found to be positively or negatively associated with prognosis in NSCLC, most of them were presented in separate studies. In our combined analysis, five different miRNAs (miR-21, miR-155, miR-*let*-7, miR-148a, and miR-148b) were evaluated in at least two selected studies using key statistics and OS data. We performed a subgroup meta-analysis of the effect of these five miRNAs on the survival of NSCLC patients as well. The meta-analysis results suggested that an elevated expression of miR-21 (*P* < 0.001) and miR-155 (*P* = 0.176) in cancerous tissue and liquid biopsy samples were associated with poor survival, whereas lower expression of miR-*let-*7 (*P* = 0.101) and miR-148a/b (*P* = 0.117 and *P* < 0.001) also predicted shorter postoperative and overall survival among the NSCLC studies [15–22, 24–29, 32–35].

The studies that used OS as a primary endpoint had high heterogeneity. This issue of heterogeneity was addressed in this study by performing a subgroup analysis. The OS, consolidated HR, and 95% CI were statistically significant in most of the studies, indicating that overexpression or underexpression of any of these miRNAs may result in a poor prognosis for NSCLC patients. Subgroup analysis based on geography revealed that the studies from America (USA) produced statistically not significant results compared to equivalent studies from Europe and Asia. These findings can conclude that miRNA expression is associated with a poorer prognosis in Asian and European NSCLC population.

Since the initial association of miR-21 with cancer in 2005, it is now considered one of the most extensively explored cancer-related miRNAs [36, 37] and may serve as a key regulator in oncogenic processes including tumor growth, migration, and invasion [38]. A growing body of evidence further supports miR-21 as a potential diagnostic and prognostic biomarker in various carcinomas [7]. Moreover, elevated miR-21 expression levels have been found associated with disease-free survival outcomes in cancer patients [8]. However, a meta-analysis by Ma et al., [9] including eight articles found no prognostic significance of miRNA-21 expression in NSCLC. Moreover, a cohort study by Voortman [20] using a large number of participants found neither predictive nor prognostic significance with miR-21 expression patterns, however, significantly associated with the age and tumor stage of the NSCLC patient's in OS. In addition, a study by Olivieri et al. [10] suggested that miRNA-21 and miRNA-155 are also upregulated in a normal person without cancer and can be associated with inflammation and senescence. Hence, there are conflicting reports as to the benefit of miR-21 as a prognostic biomarker in cancer. However, a series of recent quantitative analysis based on published studies did, in fact, suggest a significant association between high miR-21 expression levels and poor survival in NSCLC patients [8, 16, 19, 24–27, 39–41]. This meta-analysis study also supported those previous results, with the pooled effect size calculated by random effect model suggesting high expression levels of miR-21 as a moderate predictor of poor OS (HR: 1.95 and with 95% CI: 1.40–2.72) in NSCLC patients.

Evidences show that miR-155 is overexpressed in various solid tumors, including breast, lung, colon, pancreatic, and thyroid [7, 11, 42], and also plays a positive role in the development of a tumor [43]. Several studies suggest promising associations between elevated miR-155 levels and prognosis in NSCLC patients [11, 41, 43, 44]. Our meta-analysis comprised nine independent studies [17–19, 31, 34] that described the significant prognostic effect of miR-155 expression on OS among NSCLC patients, except Voortman [20], who suggested no significant association. Our combined result also supported the evidence from most of the previous studies suggesting that the high miR-155 expression is likely to result in unfavourable outcomes in NSCLC patients.

MiR-*let*-7 is considered as a protective miRNA that is downregulated in various cancers including lung cancer [29, 45, 46]. Previous studies have described that low expression of miR-*let*-7 is significantly associated with a poor prognosis in NSCLC [21, 28, 29, 32, 46, 47]. Similarly, our study also found an association between low miR-*let*-7 expression levels and a poor prognosis in NSCLC studies.

Additionally, another two downregulated miRNAs (miR-148a and miR-148b) were meta-analysed separately for the first time for NSCLC prognosis. Various studies have described the significant association of miR-148a/b overexpression level to the enhanced OS outcome among NSCLC patients [22, 33–35, 48, 49]. Less heterogeneity was observed with either of these two miRNAs in our study. The fixed effect model pooled significant HR values for the downregulation of both miRNAs that further suggested a significant prognostic role in NSCLC.

Some limitations must be considered when interpreting the results of this current study. First, the number of studies available was limited. More studies based on the prognostic role are needed to further strengthen these associations. Secondly, significant heterogeneity was observed in some of the studies, likely due to the differences in patient's clinicopathological characteristics (ethnicity, nationality, gender, age, tumor stage, and tumor grade) and different assay methods, cut-off values for the miRNA expression levels, sample preparation methods (i.e., paraffin-fixed, formalin-fixed, freshly frozen tumors, or liquid biopsy samples), follow-up durations, and key statistic parameters available. Thirdly, circulating biomarkers are more valuable and reliable than tissue biomarkers as they can be assayed before surgery and monitored throughout the tumor progression. Hence, more liquid biopsy sample-based studies need to be included. Lastly, a significant publication bias among the studies may have influenced the overall outcome. Some miRNAs that were chosen empirically or without clear justification in studies could have led to imprecise outcomes. The number of statistically insignificant studies [16, 20, 23, 24] was 15.6% (5 out of 32) and may have limited the statistical power. Patient age could be another variable that might have contributed towards heterogeneity, as four out of the 32 studies [16, 22, 50, 51] showed significant association of age with OS in NSCLC patients. Therefore, the selection of standardized protocol-based studies may likely improve the quality of such analysis. Even though there were heterogeneity, biases, and other limitations, there is growing evidence for the remarkable potential of miRNAs as prognostic biomarkers in NSCLC. More studies should be undertaken in the future to evaluate the prognostic value of specific miRNAs in serum. Large-scale and standard investigations may provide a better understanding of the mode of action and the miRNA targets, to give further insight into the use of miRNAs in lung cancer prognosis, ultimately leading to greater clinical application outcomes.

## 5. Conclusions

Several miRNAs are established to play critical roles in the initiation and development of NSCLC by functioning either as oncogenes or as tumor suppressor genes. Global miRNA expressions analysed from tumor specimens and liquid biopsy samples from patients may have a clinical relevance to serve for diagnosis, prognosis, and therapeutic outcomes in NSCLC. Our meta-analysis, representing a quantified synthesis of all published studies suggests that specific miRNA signatures which are up- or downregulated in NSCLC are associated with the poor OS and have potential prognostic and predictive value. However, large-scale standardized protocol-based studies are required to improve the accuracy and reduce the bias.

## Figures and Tables

**Table 1:** Quality Assessment of the selected studies for systemic review and meta-analysis.

Criteria	Unsatisfactory	Satisfactory	Good
Sample size	Less than ten samples	10–100 samples	>100 samples
Cox regression analysis	Only *P* value is given	Only multivariate analysis with HR, CI, and *P* value	Both univariate and multivariate analysis
Survival	Only DFS	OS, DFS	OS, DFS, RFS
miRNAs regulation	No significant upregulation or downregulation	At least one miRNA upregulated or downregulated	>1 miRNA upregulated or downregulated
Total studies	0	12	15

DFS: disease-free survival; OS: overall survival; RFS: recurrence-free survival; HR: hazard ratio; CI: confidence interval; miRNA: microRNA.

**Table 2:** Main characteristics of the studies included in the meta-analysis.

Study	Population	Study period	Sample number (patient/control)	Source of miRNA	miRNA expressed	Cancer type/subtype	Histological stage	miRNA analysis platform	Follow-up
Chen et al., 2017 [22]	China	Jul 2004–Jun 2009	165/165	Tissue (FFPE)	miR-148a	SCC: 45.5%; AC: 54.5%	T1 = 34; T2 = 89; T3 = 41; U = 1	ISH	5 yrs
Liu et al., 2017 [52]	China	2003–2005	87	Tissue (FFPE)	miR-29c	AC	T1 = 10; T2 = 62; T3 = 15	miRNA array/qRT-PCR	5 yrs
Chen et al., 2016 [23]	China	Jan 2010–Jun 2012	134	Tissue (FFPE)	miR-200a	SCC: 41.8%; AC: 48.5%; ASC: 5.2%; LCC: 4.5%	T1 = 34; T2: 26; T3a: 74	ISH	28 months (1–58)
Wang et al., 2016 [34]	China	Jan 2014–May 2015	39/39	Tissue	miR-148b	SCC: 38.5%; AC: 61.5%	T1 = 12; T2, T3 & T4 = 27	qRT-PCR	(2–60) months
Shang et al., 2016 [53]	China	Jun 2009–Sept 2010	139/139	Tissue	miR-383	SCC: 59%; AC: 41%	T1 & T2 = 63; T3 = 76	qRT-PCR	60 months
Chen et al., 2015 [50]	China	Mar 2007–Apr 2013	137/137	Tissue	miR-153	SCC: 49.6%; AC: 50.4%	T1 & T2 = 104; T3 & T4 = 33	qRT-PCR	5 yrs
Ge et al., 2015 [35]	China	May 2007–Apr 2012	151/151	Tissue	miR-148b	SCC: 46.4%; AC: 43.7%	T1 & T2 = 91; T3 & T4 = 60	qRT-PCR	N/A
Skrzypski et al., 2014 [30]	Poland	2001–2012	134	Tissue	miR-662/miR-192/miR-192^∗^	SCC	T1a = 29; T1b = 65; T2a = 4 & T2b = 31 & T3a = 5	miRNA array/qRT-PCR	5.8 yrs (4.1–10.0)
Yu et al., 2014 [13]	China	May 2008–Jul 2010	164/164	Serum	miR-375	SCC: 26.8%, AC: 60.98%, Others: 12.2%	T1 = 3; T2 = 42; T3 = 58; T4 = 34	qRT-PCR	24 months (0–56)
			53/53	Serum	miR-375	N/A	N/A	qRT-PCR	24 months (0–56)
Xiao-chun et al., 2013 [19]	China	2001–2007	60/60	Tissue	miR-21	NSCLC	T1 & T2 = 29; T3 & T4 = 31	qRT-PCR	N/A
Sanfiorenzo et al., 2013 [17]	France	Mar 2008–Mar 2010	52/20	Plasma	miR-155	SCC: 48%; AC: 52%	T1a = 8; T1b = 14; T2a = 5; T2b = 8; T3a = 7	qRT-PCR	N/A
Chen et al., 2013 [33]	China	Feb 2008–Dec 2009	47	Tissue (FFPE)	miR-148a	SCC: 52.1%; AC: 47.9%	T1 = 25; T2 & T3 = 23	qRT-PCR	N/A
Jang et al., 2012 [14]	USA	Jan 1997–Sep 2008	56/56	Tissue (FF)	miR-708	AC	N/A	miRNA array/qRT-PCR	N/A
			47/47	Tissue (FFPE)	miR-708	AC	N/A	miRNA array/qRT-PCR	N/A
Liu et al., 2012 [24]	China	2003–2005	70/70	Tissue	miR-21	AC	T1 = 10; T2 = 62; T3 = 15	miRNA array/qRT-PCR	2 yrs
Li et al., 2012 [51]	China	Jan 2006–Dec 2007	96/96	Tissue	miR-375	SCC: 43.75%; AC: 56.25%	T1 or T2 = 66; T3 = 30	qRT-PCR	30 months (8–69)
Tan et al., 2011 [31]	China	2000–2002	60/60	Tissue	miR-31	NSCLC	T1 = 21; T2 = 17 & T3 = 22	miRNA array/qRT-PCR	N/A
Donnem et al., 2011 [15]	Norway	1990–2004	191	Tissue (SCC)	miR-155	SCC	N/A	ISH	86 months (48–216)
			95	Tissue (AC)	miR-155	AC	N/A	ISH	86 months (48–216)
Saito et al., 2011 [16]	USA	1987–2009	89/89	Tissue	miR-21/miR-155	AC	T1 = 57; t2 = 22; T3 = 10	qRT-PCR	5 yrs
	Norway	1988–2003	37/37	Tissue	miR-21/miR-155	AC	T1 = 21; T2 = 5; T3 = 11	qRT-PCR	5 yrs
	Japan	1998–2008	191/191	Tissue	miR-21/miR-155	AC	T1 = 142; T2 = 49	qRT-PCR	5 yrs
Wang et al., 2011 [18]	China	2003–2005	88/17	Serum	miR-21	SCC: 23.9%, AC: 42%, Others: 34.1%	T1, T2 = 47; T3 = 41	miRNA array/qRT-PCR	52.16 months (1–73)
Gao et al., 2011 [25]	China	Feb 2004–Jan 2005	30/30	Tissue	miR-21	SCC	T1 = 17; T2 = 12 & T3 = 13	miRNA array/qRT-PCR	4-5 yrs
Gao et al., 2010 [26]	China	Apr 2008–Sep 2008	47/47	Tissue	miR-21/miR-181a	SCC: 55.32%; AC: 44.68%	T1 = 22; T2 = 12 & T3 = 13	miRNA array/qRT-PCR	N/A
Voortman et al., 2010 [20]	USA	N/A	697/79	Tissue	miR-155/miR-21/miR-*let-7/*miR-29b/miR-34a/b/c	NSCLC	N/A	qRT-PCR	8 yrs
Raponi et al., 2009 [28]	USA	Oct 1991–Jul 2002	61/10	Tissue	miR-155/mir-146b	SCC	T1: 33; T2–T4 = 28	qRT-PCR	3 yrs
Markou et al., 2008 [27]	Greece	2004–2005	48/48	Tissue	miR-21	SCC: 47.9%; AC: 52.1%	T1 & T2 = 32; T3 & T4 = 16	qRT-PCR	39 months
Yu et al., 2008 [21]	Taiwan	Sept 2000–Dec 2003	112	Tissue	miR-*let*-7	N/A	N/A	miRNA array/qRT-PCR	N/A
Yanaihara et al., 2006 [29]	USA	N/A	104/104	Tissue	miR-*let*-7/miR-155	SCC: 37.5%; AC: 62.5%	T1 = 65; T2 = 17; T3 & T4 = 22	qRT-PCR	N/A
Takamizawa et al., 2004 [32]	Japan	N/A	143	Tissue	miR-*let*-7	SCC: 17.5%; AC: 73.4%; LCC: 6.3%; ASC: 2.8%	T1 = 71; T2 = 19 & T3 = 49	qRT-PCR	N/A

FF: formalin-fixed; FFPE: formalin-fixed paraffin-embedded; AC: adenocarcinoma; SCC: squamous cell carcinoma; LCC: large-cell carcinoma; ASC: adeno-squamous carcinoma; NSCLC: non-small-cell lung carcinoma; qRT-PCR: quantitative real time PCR; N/A: not available.

**Table 3:** MicroRNA regulation (upregulated and downregulated) reported from the selected studies.

Consistently reported upregulated and downregulated miRNAs in selected studies					
Upregulated miRNAs			Downregulated miRNAs		
miRNA	Number of studies (reference)	Number of samples	miRNA	Number of studies (reference)	Number of samples
miR-21	8 [16, 18–20, 24–27]	1871	miR-*let*-7	5 [18, 20, 21, 29, 32]	1445
miR-155	6 [15–17, 20, 28, 29]	2176	miR-30a	4 [14, 25, 26, 31]	380
miR-182	5 [14, 18, 21, 28, 31]	709	miR-126	4 [25, 26, 28, 29]	433
miR-210	4 [14, 28, 29, 31]	605	miR-181a	3 [18, 25, 26]	242
miR-31	4 [14, 25, 26, 31]	480	miR-143	3 [25, 26, 29]	362
miR-191	3 [17, 28, 29]	383	miR-486-5p	2 [14, 31]	226
miR-205	3 [18, 27, 29]	392	miR-375	2 [13, 51]	313
miR-200a	2 [23, 28]	339	miR-148a	2 [22, 33]	426
miR-412	2 [25, 26]	154	miR-34b	2 [14, 20]	845
miR-135b	2 [14, 26]	300	miR-148b	2 [34, 35]	380
miR-34a	2 [20, 25]	699	miR-29c	2 [25, 52]	234
miR-192	2 [29, 30]	342	miR-29a	2 [25, 26]	154

**Table 4:** The pooled associations between different subgroups and prognosis of patients with NSCLC.

Subgroup	Number of patients	Number of studies	HR (95% CI)	*P* value	Heterogeneity	
					I-square	*P*
Overall effect	5553	32	1.59 (1.39–1.82)	<0.001	84.97%	<0.001
*MicroRNAs*						
miR-21	2025	10	1.95 (1.40–2.72)	<0.001	88.05%	<0.001
miR-155	2047	9	1.33 (0.87–2.04)	<0.001	73.27%	<0.001
miR-*let*-7	1239	4	1.94 (0.87–4.32)	0.101	87.59%	<0.001
miR-148a	377	2	2.33 (0.80–6.71)	0.117	57.92%	0.123
miR-148b	380	2	2.28 (1.64–3.17)	<0.001	0.00	0.749
*miRNA assay method*						
ISH	750	4	1.05 (0.58–1.87)	0.870	75.51%	0.007
Microarray/qRT-PCR	4803	28	1.63 (1.42–1.87)	<0.001	85.49%	<0.001
*Analysis type*						
Multivariate	4785	28	1.54 (1.35–1.76)	<0.001	84.81%	<0.001
Univariate	768	4	2.29 (1.02–5.12)	0.043	72.34%	0.013
*Source of miRNA*						
Paired Tissue	3999	20	1.67 (1.39–1.99)	<0.001	83.98%	<0.001
Blood	611	4	1.73 (1.13–2.65)	0.012	68.02%	0.025
*Patient origin*						
Asia	3432	20	2.05 (1.66–2.53)	<0.001	78.85%	<0.001
Europe	662	6	1.24 (1.05–1.47)	0.011	61.28%	0.006
USA	1439	6	2.26 (1.81–2.83)	<0.001	0.00	0.946

ISH: *in situ* hybridization; qRT-PCR: quantitative real-time polymerase chain reaction; HR: hazard ratio; CI: confidence intervals.

## Supplementary Materials

Supplementary Materials The scientific literature published from January 2004 to March 2017 was interrogated using 5 different search engines: Scopus, PubMed, Science Direct, Web of Science, and Medline using key search words, including “microRNA expression or miRNA expression,” “lung cancer or NSCLC,” “prognosis,” “radiotherapy,” “radioresistance,” “radiosensitivity,” and “Human”. Literature search results are noted in Supplementary Table 1.
