# Supplementary material for: Prognostic Role of MicroRNAs in Human Non-Small-Cell Lung Cancer: A Systematic Review and Meta-Analysis
Source: Dis Markers. 2018 Oct 21;2018:8309015. doi: 10.1155/2018/8309015 (PMC6260404; doi:10.1155/2018/8309015)
Supplement: Supplementary Materials — The scientific literature published from January 2004 to March 2017 was interrogated using 5 different search engines: Scopus, PubMed, Science Direct, Web of Science, and Medline using key search words, including “microRNA expression or miRNA expression,” “lung cancer or NSCLC,” “prognosis,” “radiotherapy,” “radioresistance,” “radiosensitivity,” and “Human”. Literature search results are noted in Supplementary Table 1. [file 8309015.f1.docx]

**Supporting Information**

Table 1: Literature search showing search number matches in different search engines.

| S. N | Keyword | Scopus | PubMed | Science Direct | Web of Science | Medline |
| --- | --- | --- | --- | --- | --- | --- |
| 1 | Lung Cancer | 184,760 | 118,564 | 181,456 | 180,135 | 1,930 |
| 2 | Radiotherapy | 338,336 | 308729 | 247, 012 | 154.739 | 3,361 |
| 3 | Radioresistance | 4,025 | 3378 | 8,426 | 3,492 | 10 |
| 4 | Radiosensitivity | 22,308 | 55,860 | 20,697 | 8,303 | 0 |
| 5 | miRNA expression | 7,081 | 6,617 | 9,741 | 6,605 | 10 |
| 6 | Prognosis | 809,403 | 1,479,387 | 528,116 | 284,533 | 5,839 |
| 1,5 | miRNA expression lung cancer | 323 | 2,254 | 8,007 | 2,471 | 18 |
| 5,1,6 | miRNA expression  lung cancer  prognosis | 419 | 578 | 987 | 651 | 8 |
